# The effects of whole body vibration in patients with type 2 diabetes: a systematic review and meta-analysis of randomized controlled trials

**DOI:** 10.1590/bjpt-rbf.2014.0133

**Published:** 2015-11-17

**Authors:** Caroline C. Robinson, Rodrigo P. G. Barreto, Graciele Sbruzzi, Rodrigo D. M. Plentz

**Affiliations:** 1 Universidade Federal de Ciências da Saúde de Porto Alegre (UFCSPA), Porto Alegre, RS, Brazil; 2 Programa de Pós-graduação em Ciências da Saúde, UFCSPA, Porto Alegre, RS, Brazil; 3 Programa de Pós-graduação em Ciências da Reabilitação, UFCSPA, Porto Alegre, RS, Brazil; 4 Curso de Fisioterapia, Universidade Federal do Rio Grande do Sul (UFRGS), Porto Alegre, RS, Brazil

**Keywords:** type 2 diabetes mellitus, exercise, physical activity, whole body vibration, blood glucose, glycemic control

## Abstract

**Background:**

Whole body vibration (WBV) has been used to increase physical activity levels in patients with type 2 diabetes mellitus (T2DM).

**Objective:**

To carry out a systematic review of the effects of WBV on the glycemic control, cardiovascular risk factors, and physical and functional capacity of patients with T2DM.

**Method:**

MEDLINE, LILACS, PEDro, and Cochrane Central Register of Controlled Trials were searched up to June 1^st^, 2015. Randomized controlled trials investigating the effects of WBV, compared to control or other intervention, on blood glucose levels, blood and physical cardiovascular risk factors, and physical and functional capacity in adult individuals with T2DM. Two independent reviewers extracted the data regarding authors, year of publication, number of participants, gender, age, WBV parameters and description of intervention, type of comparison, and mean and standard deviation of pre and post assessments.

**Results:**

Out of 585 potentially eligible articles, two studies (reported in four manuscripts) were considered eligible. WBV interventions provided a significant reduction of 25.7 ml/dl (95% CI:-45.3 to -6.1; I^2^: 19%) in 12 hours fasting blood glucose compared with no intervention. Improvements in glycated hemoglobin, cardiovascular risk factors, and physical and functional capacity were found only at 12 weeks after WBV intervention in comparison with no intervention.

**Conclusion:**

WBV combined with exercise seems to improve glycemic control slightly in patients with T2DM in an exposure-dependent way. Large and well-designed trials are still needed to establish the efficacy and understand whether the effects were attributed to vibration, exercise, or a combination of both.

## Introduction

Physical activity plays an important role in prevention and control of type 2 diabetes mellitus (T2DM) and its related complications^1^. Both aerobic and resistance training improve insulin action and can assist with the management of blood glucose levels, lipids, blood pressure, cardiovascular risk, mortality, and quality of life; however, exercise must be undertaken regularly for continued benefits^1^^,^ 2. Nevertheless, most of people with T2DM are not active, mirroring the inertia of a lifetime of habits and motivational barriers such as lack of interest, lack of time, and depression^3^. In addition, physical disabilities and perceived discomfort when exercising are challenges to adherence to physical activity^3^^,^ 4.

Physical therapists are able to help people plan an individualized exercise program in order to maintain good blood glucose and achieve optimal weight^5^. To help people with diabetes improve their quality of life, physical therapists can intervene with physical treatment techniques such as manual or manipulative treatments, electrophysical agents, and mechanical agents^5^^,^ 6.

Among the alternatives aimed to increase overall physical activity, whole body vibration (WBV) has been shown to be a new effective option in healthy subjects and individuals with several health conditions^7^. It is assumed that vibration activates muscle spindles and evokes muscle contractions induced by a complex spinal and supraspinal neurophysiological mechanism known as tonic vibration reflex, allowing muscular activity enhancement even in static positions^8^.

Some systematic reviews^9^^-^ 14 summarized the effects of WBV in some outcomes of specific populations as follows: improvements in bone mineral density in postmenopausal women^9^; leg muscle strength^10^ and balance improvement in older individuals^11^; balance, gait, and proprioception improvement in individuals with neurological conditions such as Parkinson's disease, multiple sclerosis, and stroke^12^; pain intensity decrease and physical function enhancement in individuals with knee osteoarthritis^13^; and functional exercise capacity and quality of life improvement in people with chronic obstructive pulmonary disease^14^. Furthermore, WBV requires significantly less time than conventional training and, therefore, reached a satisfactory compliance in previously inactive patients^11^.

Nevertheless, the effects of WBV in patients with T2DM were infrequently reported through a case report^15^ and acute^16^, crossover^17^, or pilot^18^^,^ 19 studies. In the last years, few randomized controlled trials were performed^20^^,^ 21 with conflicting results. To summarize the current evidence, we aimed carry out a systematic review of the effects of WBV intervention on the blood glucose levels, blood and physical cardiovascular risk factors, and physical and functional capacity of individuals with T2DM in comparison with a control or other intervention group.

## Method

This systematic review was performed in accordance with the Cochrane Handbook for Systematic Reviews of Interventions^22^ and the recommendations of the Brazilian Journal of Physical Therapy tutorial^23^. The protocol of this systematic review was prospectively registered at PROSPERO under the identification CRD42014010495 and can be assessed online^24^.

### Data sources and searches

Comprehensive literature searches were performed on the following electronic databases (from inception to June 1^st^, 2015): MEDLINE (accessed by PubMed), LILACS, Physiotherapy Evidence Database (PEDro), and Cochrane Central Register of Controlled Trials (Cochrane CENTRAL). The search terms included 'Whole body vibration', 'Diabetes' MeSH and synonyms, and a string of terms to optimize randomized controlled trial searches on PubMed^25^. In order to improve sensitivity, outcomes were not included in the search strategy. The references list of the articles identified in these searches were used as an additional source to identify other potentially eligible trials. The search strategy used on PubMed database can be fully assessed online^26^.

Randomized controlled trials were considered eligible if they addressed the effects of WBV on blood glucose levels, blood and physical cardiovascular risk factors, and physical and functional capacity in adult patients with T2DM, with a minimum of four weeks intervention and at least a control group not performing WBV. We considered as the primary outcome blood glucose levels, assessed by 12-hours fasting blood glucose (12-h FBG) or glycated hemoglobin (HbA1c). The secondary outcomes were blood and physical cardiovascular risk factors (blood cholesterol and triglycerides, atherogenic index, body mass index, body composition, weight, waist circumference, waist to hip ratio, blood pressure, or heart hate) and physical and functional capacity (maximal oxygen uptake, six minute walk test (6MWT) distance, muscle strength, or static and dynamic postural balance). The exclusion criteria were studies that included individuals with stated diabetic complications (e.g. diabetic peripheral neuropathy, retinopathy, or nephropathy) and studies with an unreliable description of WBV.

### Study selection

Two independent reviewers screened the titles and abstracts of all studies identified through the search strategies. A standard screening checklist based on the eligibility criteria was used for each study. Studies that did not meet the eligibility criteria, according to titles or abstracts, were excluded. The two independent reviewers retrieved full text versions of the remaining studies for a second review. There were no disagreements between reviewers.

### Data extraction and quality assessment

Two reviewers independently extracted the data from the eligible studies by using a standardized data extraction form. The following data were extracted: authors; year of publication; number of individuals analyzed; gender; age; parameters of WBV and description of intervention; type of comparison; mean and standard deviation of pre and post assessments of each outcome available. From articles referred to the same participants, the article with the larger sample was considered and the article with the smaller sample was excluded if outcome measurements were duplicated. There were no disagreements between reviewers. HbA1c and 12-h FBG mean and standard deviation values were not available in one published study^14^, but the authors informed these estimates by email.

The studies were assessed regarding methodological quality and statistical reporting using the PEDro scale^27^. When methodological quality assessment was not available on the PEDro database, two reviewers performed the ratings using the Brazilian Portuguese version of the PEDro scale^27^ items. In addition, the quality of each article was evaluated based on the recommendation of the International Society of Musculoskeletal and Neuronal Interactions (ISMNI)^28^ for reporting WBV intervention studies, consisting of 13 minimal reporting items about the WBV parameters and participant positioning. The instruments were rated independently by two reviewers. There were no disagreements between reviewers.

### Data synthesis and analysis

After data extraction, if the outcome values could not be transformed into a common numeric scale for quantitative synthesis, a descriptive synthesis was performed. For quantitative synthesis, pooled-effect estimates were obtained by comparing the change from baseline to study end for intervention and control group. The procedures for estimation of missing data^22^ were performed to obtain the standard deviation difference. Results were presented as weighted mean difference (WMD) with their respective 95% confidence intervals (CI). Meta-analysis was performed using the random effects model. The statistical heterogeneity among studies was assessed using Cochran's Q test and the inconsistency I^2^ test, in which values above 25% and 50% were considered as indicatives of moderate and high heterogeneity, respectively. Sensitivity analysis was not possible given the number of available studies, therefore when I^2^>25%, meta-analysis was not considered. A p value lower than 0.05 was considered statistically significant. All analyses were conducted using Review Manager, version 5.2.

## Results

### Description of studies

**Figure 1.**
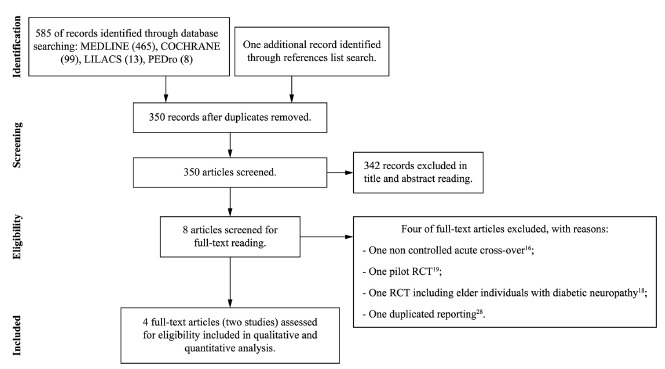
Flow diagram of studies included.

The search strategy yielded 585 articles. From these, eight^16^^,^ 19 ^-^ 21 ^,^ 29 ^-^ 32 were considered as potentially relevant and retrieved for a detailed analysis. After full text reading, four articles were excluded. As three articles^21^^,^ 31 ^,^ 32 referred to the same original study (clinical trial register: ACTRN12613000021774), they were considered as a single study. From this, two studies reporting outcomes on four different articles^20^^,^ 21 ^,^ 31 ^,^ 32 were included in this systematic review. Figure 1 shows the flow diagram of the studies and Table 1 summarizes their characteristics.

**Table 1.** Characteristics of the included studies.

**Author, year**	**Follow up**	**Participants**	**Intervention group / Comparison group**	**GenderIG %/CG %**	**Age in yearsmean (sd)IG/CG**	**Description of intervention**	**Description of comparison**	**Outcomes**	**Results**
Behboudi et al.^20^, 2011	8-wk	T2DM diagnosis, males, < 250 mg/dl 12-h FBG, non-smoking or in regular exercise programs.	WBV + Aerobic exercise (AE): 10; / AE: 10; control (C): 10.	WBV: 100 (M) / AE: 100 (M) C:100 (M)	WBV: 49.20 (3.94); / AE: 53.10 (6.57); C: 52.30 (6.17)	30 to 60 min of increasing aerobic program plus 8 to 24 min (110° squat positioning) on a vibrator (2 mm amplitude; 30 Hz; 1 min vibration and 1 min of rest).	AE: 30 to 60 min of increasing aerobic program; C: keep routine activities.	VO_2max_ (one mile walk test); BMI; %BFM (caliper and Sirri formula); insulin, 12-h FBG, HbA1c (cubital blood).	After 4 and 8 weeks of exercise, VO_2max_ significantly increased only in AE group. BMI, %BFM, 12h-FBG, HbA1c, and insulin did not change significantly in AE or WBV groups. 12h-FBG was significantly higher in C group than post intervention WBV and AE groups.
Del Pozo-Cruz et al.^31^, 2013	12-wk	T2DM diagnosis by ADA criteria, HbA1c < 10%, not receiving physical therapy.	WBV + exercises: 19 /usual-care control group (C): 20.	WBV: 55 (M); 45 (F) / C: 50 (M); 50 (F)	WBV: 71.60 (8.54) / C: 66.80 (10.83)	Eight upper and lower limb exercises with progressive 30 to 60-s duration (30-s interval between them) on an oscillating platform (1 to 2g; 12 to 16Hz; 4mm peak to peak amplitude) in a squat position with 100° knee flexion.	C: Keep nutritional and exercise habits.	TUG; Postural sway on the Wii Balance Board (WBB): AP and ML CoP excursion with eyes open (EO) and closed (EC), feet apart (FA) and together (FT).	After 12 weeks, significant between-group differences in CoP excursions with EC (FA and FT) were found. Participants in the WBV group exhibited significantly lower CoP excursions with EC after the intervention, while participants in the control group experienced a non-significant greater excursion with EO (ML). No significant difference in the TUG values post intervention.
Sañudo et al.^21^, 2013	12-wk	The same participants of Del Pozo-Cruz et al.^31^	WBV group (WBV): 20 /usual-care control group (C): 20.	WBV: 55 (M); 45 (F) / C: 50 (M); 50 (F)	WBV: 72 (8) / C: 67 (11)	WBV: description on study Del Pozo-Cruz et al.^31^	C: Keep nutritional and exercise habits.	Body composition [waist circumference, waist-to-hip ratio, weight, height, %BFM] heart rate, and blood flow [femoral artery diameter, maximum systolic velocity, maximum diastolic velocity, time averaged mean, pulsatility index and resistance index, mean velocity, and peak blood velocities].	After a 12-wk WBV intervention, weight, waist circumference, waist-to-hip ratio, %BFM, blood flow, and maximum diastolic velocity improved significantly compared to C group. Mean velocity, maximum diastolic velocity, and peak blood velocities showed significant differences within-WBV analysis.
Del Pozo-Cruz et al.^32^, 2014	12-wk	The same participants of Del Pozo-Cruz et al.^31^	WBV group (WBV): 19 /usual-care control group (C): 20.	WBV: 55 (M); 45 (F) / C: 50 (M); 50 (F)	WBV: 71.60 (8.54) / C: 66.80 (10.83)	WBV: description on study Del Pozo-Cruz et al.^31^	C: Keep nutritional and exercise habits.	HbA1c; 12-h FBG; Cholesterol, triglycerides, atherogenic index, high density lipoprotein (HDL) and low density lipoprotein (LDL); TUG; 6MWT distance; 30s-STS test. Feasibility, adherence, compliance, and safety.	HbA1, 12-h FBG, cholesterol, triglycerides, and atherogenic index significantly decreased in WBV group compared to C group. No significant changes were detected for HDL, LDL, and LDL/HDL as well as TUG. 6MWT distance and 30-s STS test significantly improved in WBV group compared to C group. There was no report of negative effects during treatment. Drop outs were due to lack of time or interest and 76% of all participants completed the 12-wk program.

Wk: week; T2DM: type 2 diabetes mellitus; 12-h FBG: 12-hour fasting blood glucose; ADA: American Diabetes Association; HbA1c: glycated hemoglobin; WBV: whole body vibration; C: control group; IG: intervention group; CG: comparison group; AE: aerobic exercise; M: male; F: female; SD: standard deviation; VO_2max:_ maximal oxygen uptake; BMI: body mass index; % BFM: percentage of body fat mass; TUG: timed up and go test; WBB: Wii Balance Board; AP: antero-posterior; ML: medio-lateral; CoP: center of pressure; EO: eyes open; EC: eyes closed; FA: feet apart; FT: feet together; HDL: high density lipoprotein; LDL: low density lipoprotein; 6MWT: six-minute walk test distance; 30s-STS: 30-second sit to stand.

A total of 70 participants with T2DM were assessed. The year of publication of the included studies ranged from 2011 to 2014. Both the studies included individuals with T2DM diagnosis and excluded those with established diabetes complications and HbA1c>10% or fasting blood glucose >250 ml/dl. Age ranged from adult to elderly classification and only males were included by Behboudi et al.^20^ while the other study^21^^,^ 31 ^,^ 32 included both genders. All of the studies randomly allocated the individuals to a control group without additional intervention, keeping normal daily activities and medical instructions. In addition, Behboudi et al.^20^ randomly allocated individuals to a third group that performed an increasing aerobic exercise (AE) program only.

Regarding WBV intervention, both studies^20^^,^ 21 ^,^ 31 ^,^ 32 applied an intermittent exposure to WBV and acceleration and frequency parameters were very similar. Studies kept peak acceleration between 1 and 2 g (units of gravity; 1g=1 m.s^-^ 2). In Behboudi et al.^20^, the peak acceleration was influenced mainly by higher vibration frequencies and lower amplitude, but in Sañudo et al.^21^ and Del Pozo-Cruz et al.^31^^,^ 32, higher amplitude and lower vibration frequencies determined the peak acceleration.

Both the studies proposed a thrice-weekly intervention of WBV with total session duration increasing progressively from 12 (8-16) to 14 (16-24) minutes. All of the studies designed protocols in which individuals stood on the vibrating platform in a 100 to 110° squat position (considering total knee extension as 180°) and the vibratory stimulus was not isolated. Behboudi et al.^20^ proposed WBV in addition to an increasing AE program (WBV+AE) with a follow-up after eight weeks. The study reported by Sañudo et al.^21^ and Del Pozo-Cruz et al.^31^^,^ 32 proposed a protocol of lower and upper limb exercises performed on the vibrating platform with a follow up after 12 weeks.

No adverse effects were reported in any of the studies. Loss of follow-up occurred only in the assessment after 12 weeks^21^^,^ 31 ^,^ 32, in which five participants from the control group dropped out because of lack of interest. Six participants from the intervention group dropped out because of lack of time (five participants) and change of home address (one participant). Participants attended more than 75% of the sessions in both trials^20^^,^ 21 ^,^ 31 ^,^ 32.

Overall, the methodological quality assessed by the PEDro scale was low to moderate (Table 2). Table 3 shows the quality of each article based on the recommendation of the ISMNI^28^ for reporting WBV intervention studies.

**Table 2.** Methodological quality assessment by the Physiotherapy Evidence Database (PEDro) Scale.

	**PEDro Scale Items**											
**Author**	**1**	**2**	**3**	**4**	**5**	**6**	**7**	**8**	**9**	**10**	**11**	**Score**
Behboudi et al.^20^	Yes	Yes	No	Yes	No	No	No	Yes	No	Yes	No	4/10*
Del Pozo-Cruz et al.^31^	Yes	Yes	No	Yes	No	No	Yes	No	No	Yes	Yes	5/10
Sañudo et al.^21^	No	Yes	No	Yes	No	No	Yes	No	No	Yes	Yes	5/10
Del Pozo-Cruz et al.^32^	Yes	Yes	No	Yes	No	No	No	No	No	Yes	Yes	4/10

1: Eligibility criteria; 2: Random allocation; 3: Concealed allocation; 4: Baseline comparability; 5: Blind subjects; 6: Blind therapists; 7: blind assessors; 8: Adequate follow up; 9: "Intention-to-treat" analysis; 10: Between-group comparisons; 11: Point estimates and variability. Eligibility criteria item does not contribute to total score. *The methodological quality assessment was performed by the reviewers.

**Table 3.** Assessment of minimum items reported for whole body vibration interventions.

	**International Society of Musculoskeletal and Neuronal Interactions Items**												
**Author**	**1**	**2**	**3**	**4**	**5**	**6**	**7**	**8**	**9**	**10**	**11**	**12**	**13**
Behboudi et al.^20^	Yes	No	Yes	Unclear	No	No	No	Yes	No	No	No	Yes	Unclear
Del Pozo-Cruz et al.^31^	Yes	Yes	Yes	Yes	Yes	No	No	Yes	Yes	No	Yes	Yes	Yes
Sañudo et al.^21^	Yes	No	Yes	No	No	No	No	Yes	No	No	No	Yes	Yes
Del Pozo-Cruz et al.^32^	Yes	Yes	Yes	Yes	No	No	No	Yes	No	No	No	Yes	Yes

1: Brand name of vibration platform; 2: Type of vibration; 3: Vibration frequency; 4: Vibration amplitude; 5: Peak acceleration; 6: Accuracy of vibration parameter; 7: Evaluation of skidding of the feet; 8: Changes of vibration parameters; 9: Rationale for choosing vibration parameters; 10: Support devices during vibration exposure; 11: Type of footwear; 12: Body position; 13: Description of exercise.

### Blood glucose levels

**Figure 2.**
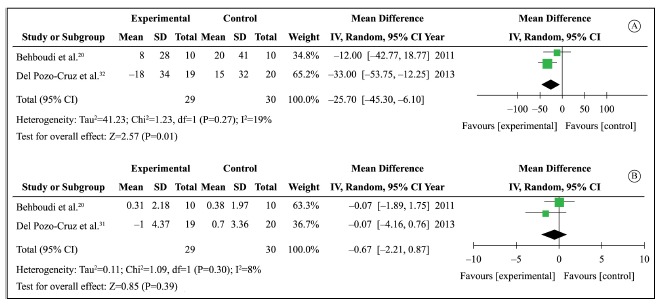
(A) The mean difference and 95% confidence interval (CI) of 12-hour fasting blood glucose in ml/dl for treatment with WBV (experimental) versus comparator (control); (B) Mean difference and 95% confidence interval (CI) of body mass index in Kg/cm^2^ for treatment with WBV (experimental) versus comparator (control).

For 12-h FBG, meta-analysis was performed and included data of two trials^20^^,^ 32 with a total of 59 patients (29 of which were on WBV). The comparison groups did not perform any intervention. There was an improvement in 12-h FBG by reduction in 25.7 ml/dl (95% CI: -45.3 to -6.1; I^2^: 19%), favoring WBV intervention (Figure 2A). There was no additional effect (p=0.09) of WBV to an eight-week increasing AE program regarding 12-h FBG, but both the groups (WBV+AE and AE only) presented significantly lower 12-h FBG levels (p=0.02) than the control group^20^.

Regarding HbA1c, a meta-analysis was not considered given an I^2^ of 80% between studies. After the 12-week program of upper and lower limb exercises performed on the vibrating platform, participants in the intervention group exhibited significantly lower levels of HbA1c (p=0.002) at the time of follow-up when compared to the control group, with a mean difference of −0.55% (95% CI: −0.15 to −0.76)^32^. The eight-week WBV+AE program was not sufficient to promote a significant difference in HbA1c levels compared to the control group. Furthermore, there was no additional effect of WBV on the eight-week AE program as no significant difference in HbA1c levels was found between WBV+AE and AE only. Both intervention groups did not differ significantly from controls.

### Blood and physical cardiovascular risk factors

Regarding secondary outcomes, a meta-analysis was only possible for Body Mass Index (BMI). Data of two studies^20^^,^ 31 with a total of 59 patients (29 of which were on WBV) were included and comparison groups did not perform any intervention. A non-significant decrease of 0.67 Kg.cm^-^ 2 (95% CI:-2.21 to 0.87; I^2^: 8%) in BMI was observed (Figure 2B).

After the 12-week program of upper and lower exercises performed on the vibrating platform, a significant decrease (p<0.050) was found in cholesterol, triglycerides, atherogenic index^32^, weight, waist circumference, waist-to-hip ratio, and body fat percentage^21^ compared to the control group. However, no statistically significant changes were detected for high-density lipoprotein (LDL), low-density lipoprotein (LDL), or LDL/HDL^32^. After the eight week WBV+AE program, no significant differences in body fat percentage were found compared to the control group or compared to the AE group^20^.

### Physical and functional capacity

Improvements (p<0.05) were found in the 6MWT distance and muscle strength assessed by the 30-second Sit-to-Stand (30s-STS) test after the 12-week WBV program with upper and lower limb exercises compared with the control group. Regarding static balance, the same comparison showed a significant decrease in center of pressure excursions with eyes closed (feet apart and together), but TUG time did not improve significantly^32^. Although maximal oxygen uptake increased significantly (p=0.01) after the eight-week WBV+AE and AE only programs, WBV had no additional effect on AE (p=0.3)^20^.

## Discussion

### Summary of evidence

It seems that the 12-week progressive intervention with WBV and exercise was sufficient for a statistically significant, but slight improvement in the 12-h FBG and HbA1c of individuals with T2DM, in comparison with no intervention. Furthermore, the eight-week intervention improved 12-h FBG, but did not improve HbA1c.

Because erythrocytes are freely permeable to glucose, the level of HbA1c in a blood sample provides a glycemic history of the previous 120 days, the average erythrocyte lifespan^33^. It is possible that a period of eight weeks was not enough to reach modifications in blood glucose profile, as no significant alterations were found in the WBV+AE or AE only programs.

The effect size for HbA1c improvement discovered after the 12-week progressive intervention with WBV and exercise was close to the one found after aerobic or resistance training reported previously in two meta analyses^34^^,^ 35. Although the vibratory stimulation was not isolated from exercises in the proposed interventions, session duration was considerably lower in the WBV studies (8 to 24 minutes) than in the aerobic or resistance training studies (40 to 75 minutes)^34^^,^ 35. This fact corroborates other studies that found similar results in WBV application compared conventional intervention, but in a shorter time of exposure^36^^-^ 38.

The meta-analysis for BMI found no significant decrease after the WBV interventions. According to Cochrane^39^, although WBV has gained popularity as a modality for weight loss, it does not have the ability to generate large energy expenditure to substitute conventional aerobic exercise. However, it had positive effects on blood flow^32^^,^ 40 that could indirectly improve associated diseases such as hypertension. In fact, this could be seen in some of the blood and physical markers of cardiovascular risk (cholesterol, triglycerides, atherogenic index, body weight, waist circumference, and waist-to-hip ratio) that improved after 12 weeks of progressive intervention with WBV combined with exercises^32^.

It seems that an eight-week WBV intervention was not enough to reach significant improvements in the aerobic capacity^20^ of patients with T2DM. In contrast, the 12-week progressive intervention with WBV and exercise improved aerobic capacity measured by the 6MWT distance, with similar values to those found in a multi-center study on fitness among healthy elderly subjects^41^. The same improvement was found in lower limb strength measured through the 30s-STS. It is possible that the time of exposure in patients with T2DM must be greater than that required for the non-diabetic population. For example, a previous meta-analysis found a significant beneficial effect of WBV on lower limb strength of elderly subjects with a treatment effect comparable to other forms of active exercises (e.g. resistance training) within six to 10 weeks^10^.

## Limitations and conclusions

This is the first systematic review to synthesize the effects of these outcomes in individuals with T2DM after WBV interventions. Analysis from data extraction of this systematic review was limited by the small number of available trials and duplicated articles. Furthermore, results from this systematic review must be interpreted with caution as most of the trials have some methodological limitations such as lack of concealed allocation and intention-to-treat analysis. Regarding the minimal items required for WBV intervention reproducibility, clear reporting is still necessary of the type of vibration, whether amplitude displacement was peak-to-peak, the peak acceleration, whether and how accuracy of vibration parameter were assessed, whether and how skidding of the feet were avoided, what was the rationale for choosing specific vibration parameters, whether and what support devices were used during vibration exposure and whether the type of footwear was controlled. Failing to report those items impairs protocol reproducibility as well as protocol comparison^28^.

Despite the slight beneficial effect of WBV intervention on glycemic control, a paramount outcome for T2DM management, caution is required in extrapolating this result to practice. First, a significant reduction in glycemic values was found in comparison with no intervention and WBV was not investigated alone, but in addition to exercise. Similar caution must be taken regarding blood markers and functional capacity. Even if WBV parameters were very similar between studies, the combined exercises differed between studies and follow-up was also distinct, which may have influenced pooled effects and heterogeneity. It is necessary to highlight that these implications should only be considered for patients with T2DM without reported complications or contraindications for WBV exposure as well as glycemic profile <10% for HbA1C or <250 mg/dl for 12-h FBG. Furthermore, it seems that effectiveness of WBV is exposure-related as the 12-week intervention presented the better results.

Similarly to other studies that used WBV as an intervention in sedentary or elderly individuals^10^, there was good adherence and compliance in the 8-week and 12-week follow-up assessments. There was similar loss of follow-up in the intervention and control groups in the 12-week WBV program related to personal reasons^21^^,^ 31 ^,^ 32. Adverse effects, such as hypoglycemia, discomfort, and musculoskeletal injuries, are highly reported in studies performing exercise interventions^34^, however they were not reported in the studies included in this systematic review^20^^,^ 21 ^,^ 31 ^,^ 32.

WBV performed close to the parameters presented in the primary studies and combined with low-level exercises seems to be a safe, feasible, and less time consuming intervention to help improve the glycemic control, cardiovascular risk markers, and functional capacity of individuals with T2DM in an exposure-dependent way compared to no intervention. However, given the methodological weaknesses of the primary studies and the heterogeneous protocols, confidence is limited on the decreasing effect of WBV on 12-h FBG. Further well-designed trials are still required to strengthen the current evidence and clarify whether the effect should be attributed to vibration, exercise, or the combination of both.
